# From the Infection to the Immunotherapy in Cervical Cancer: Can We Stop the Natural Course of the Disease?

**DOI:** 10.3390/vaccines8040597

**Published:** 2020-10-10

**Authors:** Daniela Luvero, Salvatore Lopez, Giorgio Bogani, Francesco Raspagliesi, Roberto Angioli

**Affiliations:** 1 Department of Gynecology, University Campus Biomedico, 00128 Rome, Italy; r.angioli@unicampus.it; 2 Department of Gynecologic Oncology, IRCCS National Cancer Institute, 20133 Milan, Italy; salvatore.lopez@istitutotumori.mi.it (S.L.); giorgio.bogani@istitutotumori.mi.it (G.B.); francesco.raspagliesi@istitutotumori.mi.it (F.R.); 3 Department of Experimental and Clinical Medicine, Magna Graecia University, 88100 Catanzaro, Italy

**Keywords:** HPV, cervical cancer, vaccine, immunotherapy

## Abstract

Cervical cancer (CC) is the second leading cause of cancer death in women aged 20–39 years. Persistent infection with oncogenic types of human papillomavirus (HPV) represents the most important risk factor for the development of cervical cancer. Three HPVs vaccines are currently on the global market: bivalent, quadrivalent, and nonavalent. The nonavalent vaccine provides protection against almost 90% of HPV-related CC. Despite availability of primary and secondary prevention measures, CC persists as one of the most common cancers among women around the world. Although CC is a largely preventable disease, management of persistent or recurrent CC no longer amenable to control with surgery or radiation therapy has not improved significantly with the progress of modern chemotherapy and disseminated carcinoma of the cervix remains a discouraging clinical entity with a 1-year survival rate between 10% and 15%. Over the last few years, there has been increasing interest in immunotherapy as a strategy to fight tumors. This article focuses on recent discoveries about the HPV vaccine and immunotherapies in the prevention and treatment of CC, highlighting the future view.

## 1. Introduction

Cervical cancer (CC) is most common cause of cancer death in women aged 20–39 years [1]. It is estimated that over 13,170 women will be diagnosed with cervical cancer in the United States in 2019 and over 4250 women will die of the disease [1]. In 1977, Zur Hausen, a German virologist, discovered human papillomavirus (HPV) infection as the major causative agent of cervical cancer [2]. Since then, it is well known that HPV infection is necessary for the development of invasive cervical cancer and persistent infection with sexually transmitted oncogenic types of HPV represents the most important risk factor for the development of CC [3]. More than 100 distinct HPV genotypes have been described and HPV type 16 and 18 are the most frequently detected regardless of the geographical origin of the patients [3]. Although CC is a largely preventable disease thanks to primary and secondary prevention, survival of persistent or recurrent CC remains poor and still represents a major health problem with a 1-year survival rate between 10% and 15% [4]. The aim of this review was to analyze the current status and the latest evidence about HPVs vaccines and immunotherapies in the prevention and treatment of CC.

## 2. Materials and Methods

We conducted a data research using MEDLINE, EMBASE, Web of Sciences, Scopus, ClinicalTrial.gov, and Cochrane Library. We searched medical reports published between 2008 and 2020 in the English language, using the current terms: “HPV vaccine”, “immunotherapies in cervical cancer”, “Quadrivalent vaccine”, “Nonavalent vaccine”. A collection of inclusion criteria were used for selection of articles from the literature: (1) clinical trials conducted on humans; (2) English language; (3) abstract available; (4) HPV vaccines. We excluded poster presentations; case reports; retrospective and case–control studies and articles written in a language different from English. Finally, all articles regarding only radiotherapy were excluded. Three reviewers independently read the abstracts of each paperwork to identify all articles to include.

## 3. Results

We decided to divided this review in three subheadings. The first section regarding the safety, the efficacy, and the therapeutic role of HPV vaccines (7 large randomized trials and 7 Phase I and II studies), the second one about the immunotherapy role (8 studies), and the last on the WHO program and future directions.

### 3.1. Prophylactic HPV Vaccines

Three HPV vaccines are currently approved: bivalent HPV (bHPV) vaccine, produced by GlaxoSmithKline plc. (Middlesex, UK) and quadrivalent HPV (qHPV) and nonavalent HPV (nHPV) vaccine produced by Merck (Rahway, NJ, USA). All three vaccines are non-infectious because they do not contain viral DNA. The bivalent HPV vaccine contains HPV types 16 and 18. The qHPV vaccine contains HPV types 6, 11, 16, and 18. The nHPV vaccine contains HPV types 6, 11, 16, 18, 31, 33, 45, 52, and 58. All three vaccines have been developed with non-infective recombinant virus-like particle (VLP) [5]. bHPV has the least antigenic concentration of the three vaccines while nHPV contains more than twice the aluminum load of qHPV and more than twice the antigenic load for HPV 16 and 18 in order to induce antibody responses [6]. In order to prevent HPV infection, HPV vaccines need to be administered, if possible, before the first exposure to virus, that is, before onset of sexual activity. All vaccines were administered in a three-dose schedule. Although the implementation of screening programs, the HPV vaccination rate varies between countries, it has been estimated that coverage rates ranged from 8% to 98% across 82 countries in 2017 [7].

#### 3.1.1. Safety

All three vaccines have been extensively tested for safety and have been fully monitored after FDA (Food and Drug Administration) approval. However, the most common AE (Adverse Event) reported were: pain, fatigue, redness, fever, GI (gastro-intestinal) symptoms (diarrhea, nausea, vomiting), headache, myalgia, arthralgia, and swelling [8,9,10,11,12,13,14,15]. Serious AE were also reported but they were considered not related to the vaccination. When nHPV and the qHPV vaccine were compared in clinical trial, adverse events were more common in the nHPV group than the qHPV group and pain in the injection site was reported as the most common adverse event [15]. In the NCT00543543, the rate of systemic adverse events was similar in the two groups (55.8% and 54.9%, respectively). SAEs (Systemic Adverse Events) were reported being 3.3% in the nHPV group and 2.6% in the qHPV group [16]. Moreover, previous reviews and meta-analysis included showed that injection site related-pain represented the most common adverse reaction and adverse events and death rate were similar between experimental and control groups [17,18]. Finally, post-licensure safety monitoring of the quadrivalent HPV vaccine (from 2009 to 2015) showed the absence of unexpected safety concerns [19]. Although the WHO stated that vaccines are safe, side effects represent the main reason why people are still doubtful regarding HPV vaccination [20]. In conclusion, HPV vaccines seem to be safe but longer follow-up is still needed to assess safety of this vaccine.

#### 3.1.2. Efficacy

Seven large, blinded, randomized, controlled trials enrolling young women aging 15–26 years evaluated the efficacy of bHPV (HPV-023, PATRICIA and the Costa Rica trials) [21,22,23], the qHPV (HPV-P-007, the FUTURE I, the FUTURE II trials) [24,25,26], and nHPV vaccine (NCT00543543) [16]. These seven studies included more than 50,000 patients and the efficacy on CIN2+ in a population never been infected by the virus was between 89.8% and 100% with the three doses schedule. Although several randomized controlled trials designed to compare the efficacy of one dose of HPV vaccine versus two- or three-dose schedules are ongoing in Costa Rica (ESCUDDO; NCT03180034), Kenya (KEN-SHE; NCT03675256), Gambia (HANDS; NCT03832049), and Tanzania (DoRIS; NCT02834637]), efficacy and immunogenicity data already exist from participants who received a single dose of HPV vaccine through clinical trials in which patients did not complete two- or three-dose schedules. Several observational studies showed that also a single dose of HPV vaccine may be effective against vaccine-type HPV [27]. A multicenter prospective cohort study, originally designed as a randomized trial to compare the immunogenicity and frequency of persistent infection and cervical precancerous lesions caused by vaccine-targeted HPV after vaccination with two doses or three doses of qHPV, was interrupted by the Indian government in April 2010. Thus, the clinical trial became a prospective observational cohort study comparing three different schedules [28]. A total of 17,729 participants were recruited, where 4348 (25%) girls received three doses, 4979 (28%) received two doses on days 1 and after 6 months or later, 3452 (19%) received two doses at days 1 and after 2 months, and 4950 (28%) received one dose. Immune response with the two-dose vaccine was non-inferior to the three-dose group at 7 months, but was inferior in the two-dose default and one-dose default groups after 18 months [28]. Consistent with these results, a non-randomized sub-analysis of the Costa Rica trial including 5967 women receiving three vaccine doses (2957 HPV vaccine vs. 3010 control vaccine), 802 receiving two doses (422 HPV vs. 380 control), and 384 receiving one dose showed that two doses of the bHPV vaccine, and maybe even one dose, were as protective as three doses [29]. After seven years following initial vaccination, 100% of enrolled patients in all dose groups remained HPV16 and HPV18 antibody positive in the blood [30]. Furthermore, Kreimer et al., combining data from the PATRICIA trial and Costa Rica vaccine, evaluated the efficacy of fewer than three doses of an HPV-16/18 vaccine [31]. They reported that one and two doses of the HPV-16/18 were as protective as three doses in women after 4 years follow-up. Finally, the Australian Technical Advisory Group on Immunization reported a non-inferior antibody responses after two doses given at 6 and 12 months in girls and boys aged 9–14 years compared to women (16–26 years) after three doses supporting the 2-dose schedule for adolescents of this age group [32]. A randomized, observational trial evaluating alternative nHPV vaccination schedules in young females in West Africa is still ongoing (NCT03832049). Although these preliminary data showed efficacy of less than three doses, randomized trials are still needed to validate these findings.

#### 3.1.3. Therapeutic Vaccines

All three prophylactic HPV vaccines are composed from the purified L1 protein and are highly immunogenic, inducing specific antibodies. On the other hand, therapeutic vaccines have been created to generate cell-mediated immunity. The E6 and E7 oncoproteins, which are functionally required for cellular transformation, are considered the ideal targets for immunotherapy of cervical cancer since they represent non-self antigenic targets. Several HPV therapeutic vaccines have been investigated, however, none of them have yet been licensed. Currently, electrosurgical excision of the transformation zone represents the main treatment of high-grade cervical intraepithelial neoplasia grade 2 or 3 (CIN2-3). However, incomplete excision or relapse can occur, and HPV transformed cells can progress to invasive cancer. Thus, the therapeutic vaccine is an unmet medical need that can eliminate malignant cells avoiding progression to invasive cancer. Although many pre-clinical models have supported proof-of-principle for immunotherapeutic targeting of E6 and E7 in HPV-associated malignancies, clinical translation has been incomplete, in part due to the restricted immunogenicity of the vaccines tested to date. One of the most promising vaccines is VGX-3100, which consists of two DNA plasmids encoding the E6 and E7 genes of HPV (Inovio Pharmaceuticals, Plymouth Meeting, PA, USA) [33].

In a Phase I trial, 18 women, previously treated for high-grade lesions (CIN2/3), received three doses of highly engineered plasmid DNA encoding HPV16 and HPV18 E6/E7 antigens followed by electroporation (EP) in a dose escalation study (0.3, 1, and 3 mg per plasmid). The vaccine was well tolerated and no study-related serious or grade 3 and 4 adverse events were reported. No dose-limiting toxicity was noted. Data showed VGX-3100 was capable of driving robust immune responses to antigens from high-risk HPV serotypes [34]. Based on these promising results, a Phase II clinical trial evaluated the efficacy, safety, and immunogenicity of VGX-3100 delivered by EP before a planned standard therapeutic resection of CIN2/3 lesions positive for HPV-16 or HPV-18 [35]. One hundred and sixty-seven patients were included in the study, 125 patients received VGX-3100, while 42 patients received placebo. In the per-protocol analysis, 53 (49.5%) of 107 VGX-3100 recipients and 11 (30.6%) of 36 placebo recipients had histopathological regression (*p* = 0.034). In the modified intention-to-treat analysis, 55 (48.2%) of 114 VGX-3100 recipients and 12 (30.0%) of 40 placebo recipients had histopathological regression (*p* = 0.034). Injection-site reactions occurred in most patients but they were not statistically significant between two groups. Only erythema was significantly more common in the VGX-3100 group (98/125, 78.4%) than in the placebo group (*p* = 0.007). Two Phase III clinical trials have been designed. The REVEAL I, a prospective, randomized, double-blind, placebo-controlled Phase 3 study to determine the efficacy, safety, and tolerability of VGX-3100 administered by intramuscular injection, followed by electroporation in adult women with CIN2 or CIN3 associated with the human papillomavirus (HPV) 16 and/or HPV-18 (NCT03185013). The enrollment was completed and results will be available in the fourth quarter of 2020. The second one is the REVEAL II, and it is a confirmatory randomized, double-blind, placebo-controlled study that is currently enrolling patients (NCT03721978).

### 3.2. Immunotherapy

Immune therapy aims to activate the body’s immune system and then improve the tumor-killing ability. The immune system can be innate or adaptive. The adaptive immune response is a humoral (antibody B cell-mediated) immunity and cell-mediated (T-cells specific) immunity. For its potentiality in the cancer treatments, several studies are still on going. The most important ability of the immune system is the discrimination between normal and abnormal cells in the body thanks to the presence of “checkpoints”. Cancer cells sometimes use these checkpoints to evade the immune system. One of the therapeutic promises against cancer are drugs that target these checkpoints. There are eight immune checkpoints [programmed cell death protein 1 (PD-1), cytotoxic T-lymphocyte anti- gen-4 (CTLA-4), T-cell immunoglobulin and mucin- domain containing-3 (Tim-3), 2B4, killer cell lectin like receptor G1 (KLRG-1), TIGIT, B- and T-lymphocyte attenuator (BTLA), and CD160]. PD-1 and CLTA-4 are the most promising immune checkpoints targeted in CC.

#### 3.2.1. Drugs That Target Ctla-4

It is known that cancers are recognized by the human immune system, which can help to suppress tumoral cells. Sometimes, tumors have poor immunogenicity, because they do not provide signals for CD28-mediated costimulation, which is necessary to activate T cells. The protein CTLA-4 is a CD28 homolog, and in addition, it has a greater affinity to B7-1 and B7-2, CD28 primary ligands on the surface of specialized antigen presenting cells. CTLA-4 is a negative regulator of T cell responses, and, if blocked, especially in its interactions with B7, the T cell responses augment and the antitumor immunity increases. Targeting immune-checkpoints has been showed to have an efficacy in the activation of immune responses against the tumor. In particular, CTLA-4, which is expressed in some lymphocytes T and acts as a type of “off switch” to keep the immune system in check. Ipilimumab is a human monoclonal IgG1 antibody against CTLA-4, allowing the body to overcome immune cancer suppression and also in cancers associated with HPV [36]. It has a half-life of 12–14 days [36]. In 2011, the FDA approved Ipilimumab for the treatment of unresectable metastatic melanoma and it also showed a good toxicity profile in patients with advanced cervical cancer but without a significant single-agent activity [37]. An ongoing Phase I clinical trial (NCT01711515) is analyzing the effect of chemo-radiotherapy and Ipilimumab in treating patients with stages IB2-IIB or IIIB-IVA cervical cancer with positive nodes [38]. Another antibody against CTLA-4, which has been recently developed, is Tremelimumab [36]. It is an IgG2 antibody, with a longer half-life of 22 days. [36]. It is not yet FDA approved, as Ipilimumab, but has completed Phase III [36]. For this reason, its proper dosing and schedule have not yet been defined, while it is already clear for Ipilimumab, 3 mg/kg every 3 weeks for four doses [36].

#### 3.2.2. Anti-PD-1 or PD-L1

PD-1 is a transmembrane protein found on T cells in the peripheral blood. PD-L1 is the receptor programmed death ligand-1 and is associated with antigen presenting cells such as dendritic and cancer cells [39,40]. When the PD-1 receptor binds PD-L1 and PD-L2, it keeps the T-cell energy and creates apoptosis [41]. When the PD-1/PD-L1 pathway is activated, it can inhibit HPV related to cervical, head, and neck squamous cancer cells. Therefore, blocking these pathways in patients with HPV-related cancers can block the immune response eventually induced by these HPV infected tumors [42]. Many researchers have examined PD-L1 expression in cervical cancer tissue and is represented in 34.4–96% of cervical cancer tissues, but it is rarely observed in histologically normal cervical tissue. According to the TCGA (The Cancer Genome Atlas) database, PD-L1 amplification was observed in 22% of patients with cervical squamous carcinoma.

The PD-1/PD-L1 antibody was recently approved by the FDA for metastatic melanoma, non-small cell lung cancer (NSCLC), head and neck, kidney and urothelial carcinomas, Hodgkin lymphoma, and microsatellite instability/mismatch repair (MMR) deficient cancers [43]. Nausea, fatigue, decreased appetite, joint pain, constipation, and diarrhea are the common side effects [44]. Furthermore, the combination of ipilimumab (anti-CTLA-4) and nivolumab (anti-PD-1) significantly enhanced efficacy in metastatic melanoma patients. Currently, there are multiple ongoing Phase I/II clinical trials evaluating the effect of anti-PD-1/PD-L1 therapy alone or in combination, in patients with advanced, recurrent, or persistent cervical cancer [45]: in CheckMate 358, a Phase I–II study, nivolumab 240 mg/kg was given every 2 weeks in virus-related tumors, including cervical cancer. The ORR (Overall Response Rate) was 26.3%, and the disease control rate was 70.8%. Grade 3/4 hyponatremia and diarrhea were observed [46]. Nivolumab in combination with ipilimumab has been investigated in advanced cervical cancer. In addition, Nivolumab is being tested in combinations with Urelumab, an anti-4-1BB/CD137 antibody (NCT01471210) and with Lirilumab, an anti-KIR antibody (NCT01714739). Other associations are OX40 immune modulator in combination with Tremelimumab, an anti- CTLA-4 antibody (NCT02205333), while a second Phase I/II trial (NCT01693562) is evaluating an anti-PD-L1 antibody (MEDI4736). FDA approved in 2019 Pembrolizumab for the treatment of women with recurrent or metastatic cervical cancer expressing PD-L1 based on the results of a Phase III trial KEYNOTE-158, in which Pembrolizumab was found to display a good clinical activity in 98 women with pre-treated advanced cervical cancer [47]. Patients received Pembrolizumab 3 times per week for 2 years or until disease progression or intolerable toxicity. The 83.7% of patients had PD-L1-positive cancers and had a longer median OS (Overall Survival) than those with negative tumors (11 months vs. 9.4 months, respectively). The overall response rate of the study was 12.2%, with a median follow-up of 10.2 months. A study showed 3 complete responses and 9 partial responses [48], where 65.3% of patients showed side effects of any grade and grade 3 or higher adverse events were found in 12.2% of women. Around 25% of patients showed immune-mediated adverse events: 2 hepatitis, 2 severe skin reactions, and 1 adrenal insufficiency [49]. The PD-L1 expression levels were also related to survival outcomes in other studies. In patients with diffuse PD-L1 expression affected by squamous cervical cancer, disease-free and survival rates were significantly poorer if compared with patients with marginal PD-L1 expression [50]. Based on these results, Pembrolizumab and Nivolumab appeared effective in recurrent and unresectable cervical cancers, although more studies are needed to validate these data.

Moreover, the combination of radiation therapy and immune checkpoint inhibitors has been attracting attention. As we know, the concurrent chemoradiation therapy (CCRT) represents the standard for treating locally advanced cervical cancer. Based on recent studies, which showed that tumor cells destroyed by radiation therapy lead to the activation of anti-tumor immunity, clinical trials with CCRT have been initiated in cervical cancer patients (Table 1).

#### 3.2.3. Adaptive T-Cell therapy

Two reports by Stevanović et al. [51,52] showed that antigen-specific T cells with a strong antitumor effect exist in cervical cancer tissue, playing an important role in CC immune surveillance. These studies showed that T cells that recognize HPV protein and mutant antigens that are present in cervical cancer tissue may have a potential role in preventing tumor progression and relapse. Thus, TIL (Tumour-Infiltrating Lymphocyte) therapy for cervical cancer might be the most effective treatment for advanced cervical cancer. Moreover, in a Phase II trial, Iovance Biotherapeutics reported 27 CC patients who received TIL infusion showed an ORR of 44% (*n* = 12), with 3 complete responses (CRs; 11.1%) and 9 partial responses (PRs; 33.3%) (reported in 2019 by ASCO meeting). Chimeric antigen receptor T cells (CAR-T) have been approved by the Food and Drug Administration (FDA) and are successfully improv- ing outcomes for hematological malignancies. Although research studies in CC are rare, one study investigated the killing effect of mesothelin-CAR-T in CC cells and achieved positive results [53]. A clinical trial combined autologous cytokine-induced killer (CIK) cell transfusion and radiochemotherapy in CC patients and showed that the application of CIK cells improved immune function and life quality [54]. Moreover, NK cell transfusion may also improve CC status [55]. In conclusion, adoptive cell transfer therapy should be given more attention in CC.

### 3.3. Outlook

Several clinical trials evaluating the role of immune checkpoint inhibitors in CC are ongoing and have previously reported in Table 1.

Clinical trials are also focusing on the use of several components of the immune system, integrating basic science with direct clinical application. As an example, the HPV DNA genome encodes for eight proteins, of which E6 and E7 are consistently retained and expressed, thus providing a tumor-specific target for immunotherapeutic intervention in cervical cancer. Santin et al. provided the first experience with dendritic cells pulsed with HPV E7 oncoprotein. Re-administration of these dendritic cells, previously activated in vitro, induced a strong and specific immune response against HPV antigens [56]. No side effects were reported. Furthermore, chimeric antigen receptor T cells (CAR-T) therapy has been approved by the U.S. FDA for certain malignancies. An ongoing clinical trial is investigating the role of CAR-T cell therapy in cervical cancer (NCT03356795). Finally, combinatorial therapy with other molecularly targeted drugs. Specifically, these studies are investigating the role of immunotherapy in combination with poly(ADP-ribose) polymerase (PARP) inhibitors that can enhance the infiltration of cytotoxic T lymphocytes and increase tumor antigens, need further investigation or in combination with and vascular endothelial growth factor (VEGF) inhibitors. These studies have shown promising preclinical results and are currently tested in clinical trials [57].

## 4. Discussion

Cervical cancer is a big health issue with high regional incidence and mortality rates in low-income countries due to the lack of screening programs and treatments availability. Among the years, the management of recurrent or advanced and metastatic cervical cancer, in which the surgery was not an option, was associated with poor prognosis, rapid deterioration of quality of life, and early death [58]. Recently, the cellular immunotherapy has become one of the best strategies to harness the immune system to fight tumors. Moreover, vaccines have been found to be highly effective in preventing infection and pre-invasive and invasive cervical, vulvovaginal, and anal disease.

Furthermore, 2020 promises to be a landmark year for cervical cancer. Considering the epidemiology data, with cervical cancer being a preventable tumor, the WHO “Global Strategy Towards the Elimination of Cervical Cancer as a Public Health Problem” is being developed with the aim of reaching the age-adjusted incidence rate of cervical cancer less than 4 per 100.000 women-years. This new WHO project proposes, for 2030, to be reached by all the countries: 90% of girls fully vaccinated against HPV by 15 years of age; 70% of women screened with a high precision test at 35 and 45 years of age; 90% of women identified with cervical disease receive treatment and care. These goals must be followed in combination, as shown by a brilliant mathematical model, to reach the desired incidence rate. This strategy builds on the WHO Director-General’s May 2018 call to action, that called all the WHO Member States to unite behind this common goal. For now, a draft has been published, while the official version is going to come up soon (https://www.who.int/docs/default-source/cervical-cancer/cerv-cancer-elimn-strategy-16dec-12pm.pdf).

## 5. Conclusions

Cancer immunotherapy represents a new therapeutic option for several cancer types, including patients with recurrent and metastatic cervical cancer. Several clinical trials are still ongoing, and nowdays, we do not have sufficient evidence of the clinical benefit in CC. Further studies may be needed to validate this potentiality, to reduce the incidence of adverse events and meantime preserve the efficacy of this therapy and in order to investigate different applications of immunotherapy.

However, combinations of anti-angiogenic agents with immune checkpoint inhibitors have been shown preclinically to generate more potential antitumor effects and might have clinical potential. On the other hand, radiation therapy has the potential to activate a systemic response rate to immunotherapy as well as increase local tumor control at the irradiated site.

New prospective studies will be needed to determine the perfect integration of immunotherapy, radiotherapy, and chemotherapy.

## Figures and Tables

**Table 1:** Ongoing studies about immunotherapy in cervical cancer.

NCT Number	Status	Interventions	Gender	Phases	Enrollment
NCT4188860	Recruiting	A combinationof anti PD-1 ab Camrelizumab and albumin bound Paclitaxel	Female	Phase 2	34
NCT04256213	Recruiting	Nivolumab and Ipilimumab	Female	Not Applicable	40
NCT03108495	Recruiting	LN-145 + Pembrolizumab	Female	Phase 2	138
NCT02635360	Recruiting	Pembrolizumab	Female	Phase 2	88
NCT03614949	Recruiting	Atezolizumab	Female	Phase 2	26
NCT04405349	Recruiting	Atezolizumab	Female	Phase 2	50
NCT03073525	Active, not recruiting	Atezolizumab	Female	Phase 2	25
NCT03277482	Recruiting	Durvalumab	Female	Phase 1	32
NCT03192059	Recruiting	Pembrolizumab	Female	Phase 2	43
NCT02725489	Active, not recruiting	Durvalumab	Female	Phase 2	13
NCT4230954	Recruiting	Cabozanitib and Pembrolizumab	Female	Phase 2	39
NCT03452332	Recruiting	Durvalumab/Tremelimumab	Female	Phase 1	18
NCT03508570	Recruiting	Ipilimumab	Female	Phase 1	48
NCT03833479	recruiting	CRT Maintenance TSR-042 (anti-PD-1 antibody)	Female	Phase 2	132
NCT03144466	Recruiting	CRT with Pembrolizumab	Female	Phase 1	26
NCT03298893	Recruiting	CRT with Nivolumab	Female	Phase 1	21
NCT01711515	Recruiting	CRT with Ipilimumab	Female	Phase 1	34
